# Endometrial Cancer: What Is New in Adjuvant and Molecularly Targeted Therapy?

**DOI:** 10.1155/2010/749579

**Published:** 2010-02-02

**Authors:** Flora Zagouri, George Bozas, Eftichia Kafantari, Marinos Tsiatas, Nikitas Nikitas, Meletios-A. Dimopoulos, Christos A. Papadimitriou

**Affiliations:** ^1^Department of Clinical Therapeutics, “Alexandra” Hospital, School of Medicine, University of Athens, Athens, Greece; ^2^Oncology Centre, Castle Hill Hospital, Hull and East Yorkshire NHS Trust, Cottingham, UK

## Abstract

Endometrial cancer is the most common gynaecological cancer in western countries. Radiotherapy remains the mainstay of postoperative management, but accumulating data show that adjuvant chemotherapy may display promising results after staging surgery. The prognosis of patients with metastatic disease remains disappointing with only one-year survival. Progestins represent an effective option, especially for those patients with low-grade estrogen and/or progesterone receptor positive disease. Chemotherapy using the combination of paclitaxel, doxorubicin, and cisplatin is beneficial for patients with advanced or metastatic disease after staging surgery and potentially for patients with early-stage disease and high-risk factors. Toxicity is a point in question; however, the combination of paclitaxel with carboplatin may diminish these concerns. In women with multiple medical comorbidities, single-agent chemotherapy may be better tolerated with acceptable results. Our increased knowledge of the molecular aspects of endometrial cancer biology has paved the way for clinical research to develop novel targeted antineoplastic agents (everolimus, temsirolimus, gefitinib, erlotinib, cetuximab, trastuzumab, bevacizumab, sorafenib) as more effective and less toxic options. Continued investigation into the molecular pathways of endometrial cancer development and progression will increase our knowledge of this disease leading to the discovery of novel, superior agents.

## 1. Introduction

Endometrial cancer is the most prevalent gynecological cancer in the Western World representing the third commonest cancer affecting women. By contrast, the incidence in the non-Western World is approximately tenfold lower [1]. The excellent prognosis of early-stage endometrial cancer renders it one of the most curable gynecological malignancies. Radiotherapy remains the mainstay of postoperative management, but accumulating data show that adjuvant chemotherapy may display promising results after staging surgery. The term staging surgery implies to hysterectomy, bilateral salpingoophorectomy, and pelvic and para-aortic node dissection with or without omentectomy. Unfortunately, the prognosis of patients with metastatic disease remains disappointing with only one-year survival commonly reported despite treatment efforts [2].

Systemic interventions play a key role in the treatment of advanced/metastatic and relapsed endometrial cancer. Progestins remain an effective option, especially for those patients with low-grade estrogen and/or progesterone receptor positive disease, some of whom achieve prolonged survival [3–14]. Platinum compounds, anthracyclines, and more recently taxanes have been developed in combination regimens, achieving response rates exceeding 50% and resulting in more than one-year survival in randomized trials [2, 15–40]. Today, the combination of doxorubicin 45 mg/m^2^, cisplatin 50 mg/m^2^, and paclitaxel 160 mg/m^2^ (TAP) [29] is considered the most effective chemotherapy regimen for advanced or recurrent endometrial cancer. A large GOG trial which is currently evaluating TAP against paclitaxel and carboplatin may at last provide conclusive data on the comparative efficacy of the less toxic nonanthracycline combination [2]. It is worth mentioning that the GOG 209 trial has closed to accrual, although results are not yet available.

Adjuvant chemotherapy using the same agents is beneficial for patients with advanced disease after staging surgery and potentially for patients with early-stage disease and high-risk factors, such as high-grade or nonendometrioid histology. Their combination with radiotherapy is still under debate. Toxicity is a point in question for endometrial cancer patients treated with chemotherapy, given their often advanced age and multiple comorbidities. Hematologic toxicity, cardiac toxicity, and neurotoxicity probably present more cause for concern, as they can increase the risk of treatment-related death or long-term disabilities. The development of less toxic regimens such as the combination of paclitaxel with carboplatin may diminish these concerns.

Our increased knowledge of the molecular aspects of endometrial cancer biology has paved the way for clinical research to develop novel targeted antineoplastic agents as more effective and less toxic options. This review article aims to present the gathering evidence of current adjuvant systemic treatment of endometrial cancer in an attempt to direct ongoing clinical research.

## 2. Adjuvant Chemotherapy

Radiotherapy (vaginal brachytherapy and/or pelvic irradiation) remains the mainstay of postoperative management, decreasing the rate of pelvic recurrences. Moreover, it is the preferred sole method of treatment for patients with high-risk and may be intermediate-risk early-stage disease [41–43]. Additionally, it is worth mentioning that trials in early-stage disease have shown decreased locoregional recurrence but no improvement in survival with radiotherapy. The use of adjuvant systemic treatment in endometrial cancer is an individualised decision based on the assessment of prognostic factors which increase the potential for relapse and distant metastasis such as stage, age >70 years, and histological characteristics (grade, serous or clear cell type, lymphovascular space invasion) [42, 44]. The anticipated benefits and risks for toxicity are also taken into the equation. Grade 3 endometrioid tumours, as well as the serous and clear cell variants, display a more aggressive behaviour than grades 1 and 2 endometrioid cancers. These high-risk types are commonly diagnosed as advanced or metastatic disease but early-stage cancers can also result in similarly unfavourable outcomes [45]. Surgical staging procedures also play a significant role, since a recurrence risk for a proportion of patients with ostensible stage I disease and high-risk histology may be underestimated [45, 46].

Although current evidence does not support the use of progestins in the adjuvant treatment of endometrial cancer [47, 48], chemotherapy may prove beneficial in patients with high-risk features. Three randomised trials comparing chemotherapy with radiotherapy for high- and intermediate-risk endometrial cancer have produced somewhat equivocal but important results (Table 1).

**Figure 1:**
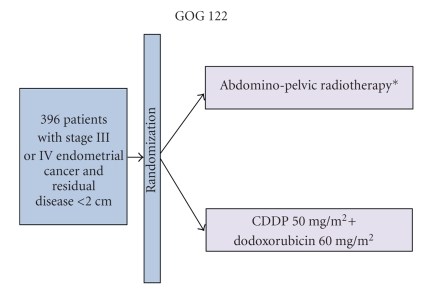
Postoperative whole abdominal radiotherapy versus combination doxorubicin-cisplatin chemotherapy in advanced endometrial carcinoma. *30-Gy in fractions with a 15-Gy boost.

Randall et al. reported the results of the GOG protocol 122 which randomized 396 women with stage III and optimally debulked stage IV endometrial cancer to postoperatively receive either whole abdominal irradiation (WAI) or chemotherapy with cisplatin and doxorubicin (Figure 1). This study favoured chemotherapy with a hazard ratio for progression of 0.71 and 0.68 for death, and five-year survival rate of 55% versus 42%, respectively [49]. Another trial included 345 intermediate- and high-risk patients (stage IC grade 3, stage II grade 3 with >50% myometrial invasion, and stage III) who were randomized to receive adjuvant chemotherapy with cisplatin, doxorubicin, and cyclosphosphamide (CAP) or external beam radiotherapy (pelvic and paraortic). Similar results in overall and disease-free survival were reported for both arms. The authors noted that radiotherapy did improve local relapse rates, while chemotherapy improved the risk for distance metastases [50]. The latest published study comparing chemotherapy with radiotherapy randomized 385 patients with >50% myometrial invasion, 61% of whom had stage I disease, to receive either CAP or external beam pelvic radiotherapy. Although the 5-year OS and PFS rates were similar in both arms, a significant improvement of PFS (HR = 0.44) and OS (HR = 0.24) was observed with chemotherapy in a subgroup comprising patients with stage IC and >70 years old, stage IC and grade 3 endometrioid tumors, stage II, and stage IIIA (positive cytology) [51]. Interestingly, no significant increase in adverse effects was observed in the CAP group versus the radiotherapy group.

Single modality adjuvant treatment with chemotherapy entails a high risk of local relapse. Indeed, 36% of the initial recurrences were limited to the pelvis in the GOG 122 study [49]. This presents a strong argument for the implementation of combined modality treatments. Nevertheless, salvage external beam radiotherapy in previously nonirradiated patients with locoregional recurrence has resulted in five-year local control rate of 54%, disease specific survival of 51%, and overall survival of 44% [42]. Consequently, radiotherapy could be considered in later stages of management rather than postoperatively with less risk for combined toxicities. In the RTOG 9708 study, 46 patients with endometrial cancer confined to the pelvis (stage I to IIIC) displaying adverse histological prognostic factors were treated with chemo-radiotherapy followed by chemotherapy with cisplatin and paclitaxel. The 5-year DFS and OS rates for stage III patients were 72% and 77%, respectively, and no relapses in stage I or II patients were recorded. However, a grade 4 long-term toxicity was reported in 4% of patients [54]. The NSGO-EC-9501/EORTC 55991 study randomized 372 patients with high-risk endometrial cancer (grade 3, deep myometrial invasion, DNA nondiploidy, serous, clear-cell, or anaplastic histology) of surgical stages I to IIIC and without paraortic lymph node involvement to receive either external beam irradiation with or without vaginal brachytherapy, or radiotherapy plus platinum-based chemotherapy. The results favoured the combined modality treatment with an HR of 0.58 for PFS [53]. Nevertheless, another recently published study of sequential chemo-radiotherapy (cyclophosphamide, cisplatin, epirubicin) versus radiotherapy alone in 157 patients, with stage IA/B, grade 3 and stages IC to IIIA of any grade, failed to show a statistically significant improvement in survival outcomes. Furthermore, chemotherapy seemed to increase intestinal toxicity requiring surgery [51].

**Figure 2:**
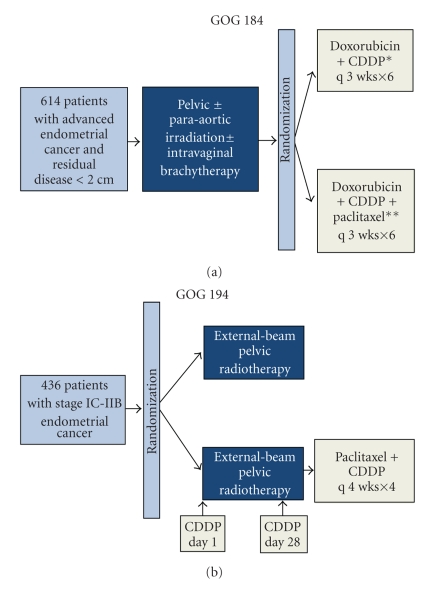
Recent randomized GOG trials of postoperative radiotherapy and/or combination chemotherapy in endometrial cancer. *Both arms received G-CSF. **Paclitaxel was administrated on day 2.

**Figure 3:**
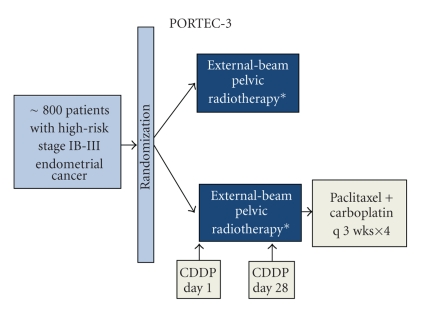
Chemotherapy and radiation therapy compared with radiation therapy alone in treating patients with high-risk stage I, stage II, or stage III endometrial cancer. *Patients with cervical involvement undergo vaginal brachytherapy.

It has become clear that questions surrounding adjuvant chemotherapy in endometrial cancer and its optimal application are far from being settled and hence form an active field of clinical research. The use of newer agents, such as paclitaxel, appears promising. Its combination with carboplatin has shown favourable efficacy and toxicity as adjuvant treatment of endometrial cancer [55] and may prove to be a valid and less toxic option to anthracycline-platinum combinations. GOG protocol 184 (Figure 2) deals with advanced stage patients randomized after surgery with optimal debulking (diameter ≤2 cm) and tumor-directed radiation to cisplatin and doxorubicin with or without paclitaxel. There was no statistically significant improvement in recurrence free survival between the two regimens. Overall, the addition of paclitaxel had little impact on recurrence free survival and was associated with increased morbidity. Of note, subset analysis revealed a 50% reduction in the risk of recurrence or death for patients with gross residual disease in the triplet arm when compared to the doublet one [56]. The advantages of identifying early-stage patients who may benefit from adjuvant chemotherapy are the subject of GOG 194 (Figure 2) and PORTEC-3 (Figure 3) trials, evaluating the addition of paclitaxel and either cisplatin or carboplatin to adjuvant radiotherapy. Notably, the aggressive nature of serous and clear cell tumours and a wealth of data from nonrandomized studies [30–41, 54, 57–67] have led many institutions to standardize adjuvant chemotherapy for all early-stage patients with such histology. However, the small volume of patients belonging to these subgroups (2%–4% of stage I endometrial cancer) hampers the design of a phase III study to further clarify the merits of this approach.

## 3. Toxicity of Systemic Chemotherapy for Endometrial Cancer and Patient Selection

Patients with endometrial cancer often present cause for concern due to toxicity, given that they are often of advanced age, with poor performance status and multiple comorbidities. The significance of age and coexisting medical conditions in clinical decision-making can be extrapolated from the observation that 40% of deaths in patients participating in clinical trials are attributed to conditions other than endometrial cancer [41]. Whilst it is true that more intensive combination regimens achieved greater efficacy in advanced or recurrent endometrial cancer, toxicity was unfortunately increased. Even among selected populations of phase II or III trials, treatment-related deaths are not uncommon despite the use of G-CSF [25–27, 29, 35]. A meta-analysis of pooled toxicity data from five randomized trials [17–19, 29, 68] comparing less intensive with more intensive chemotherapy showed that treatment intensification resulted in higher rates of severe (grades 3 and 4) nausea and vomiting, gastrointestinal toxicity, thrombocytopenia, infection, renal toxicity, and neurotoxicity with odds ratios of 2.73, 2.48, 4.44, 4.36, 3.55, and 5.81, respectively [15].

Toxicity far outweighs any concerns in the adjuvant setting. In the phase III trial comparing WAI with AP chemotherapy for stage III/IV endometrial cancer after staging surgery, 13 treatment-related deaths were reported among 396 randomized patients, most of which involved the chemotherapy arm. Severe hematologic toxicity was documented in 88% of patients on AP as opposed to 14% of those on WAI arm [49]. Furthermore, 17% of patients receiving AP discontinued treatment due to toxicity, compared to only 3% treated with WAI. Maggi et al. [50] reported a rate of 35% for grades 3 and 4 neutropenia in stage IC-III patients receiving adjuvant CAP as opposed to a 16% rate of severe gastrointestinal toxicity in patients treated with radiotherapy [50]. Gastrointestinal toxicity may be more frequently observed in patients receiving both radiotherapy and chemotherapy, as reported by Kuoppala et al. [51], where the addition of the cyclophosphamide, epirubicin, and cisplatin (CEP) regimen to pelvic irradiation increased the rate of gastrointestinal complications requiring surgery from 2.7% to 9.5%.

Since previous pelvic irradiation depletes the hematopoietic stem cell pool and increases the potential for severe hematologic toxicity, such patients have habitually received lower doses of chemotherapy in phase III trials [17, 29, 35]. Albeit G-CSF support may improve the tolerability of doublet and triplet regimens, grade 4 neutropenia remains notably frequent at over 35% [29, 35].

Elderly patients with predisposing factors or preexisting cardiac disease about to be treated with anthracycline based chemotherapy are a source of concern for cardiotoxicity. Patients enrolled in studies are routinely screened for left ventricular function defects and those with preexisting dysfunction or active coronary heart disease are typically excluded [17–19, 23, 29]. Finally, patients with longstanding diabetes mellitus may be more prone to neurotoxicity, a debilitating condition commonly associated with combinations including cisplatin and/or paclitaxel [20–22, 26].

The use of prophylactic G-CSF, as well as the use of single-agent chemotherapy or less toxic regimens, such as the combination of carboplatin with paclitaxel or carboplatin with liposomal doxorubicin, is reasonable options to be considered for improved tolerability.

Overall, research data should be interpreted with caution. Populations treated in studies are very likely to differ from the average population encountered in common clinical practice; quality of life factors must be considered in the individualization of management decisions. 

## 4. Targeted Therapy for Endometrial Cancer

### 4.1. Genetic Alterations in Endometrial Cancer

Nowadays, we have a better understanding of molecular characteristics of endometrial cancer which seem to concur with previously established clinical and histological disease types (Table 2) [69–79]. The importance of angiogenesis has been recognised in regard to the natural history of endometrial cancer and presents potential clinical and therapeutic implications. Tumor suppressor protein PTEN (phosphatase and tensin homolog deleted on chromosome ten), a lipid-protein phosphatase key to the regulation of normal cell function, has been reported to be altered in up to 83% of endometrioid carcinomas [76, 80, 81]. PTEN inactivation is most commonly caused by mutations in both alleles resulting in the complete loss of function (reviewed in [76, 80]). It principally targets and dephosphorylates 3, 4, 5-trisphosphoinositides resulting in the inhibition of the phosphatidylinositol-3 kinase (PI3K) pathway [82]. Total lack or impairment of PTEN protein from cancer cells causes hyperactivation of the PI3K pathway, leading to uncontrolled function of several kinases, including the serine/threonine kinase mTOR (mammalian target of rapamycin) (reviewed in [80]). PI3KCA mutation is seen in 36% of endometrioid endometrial cancers and is most common in tumors that also bear the PTEN mutation [83]. Additionally, the upregulation of proapoptotic mechanisms involving AKT-dependent mechanisms is mediated through PTEN, as is the downregulation of antiapoptotic mechanisms through Bcl-2 [84]. Since the protein phosphatase activity of PTEN is involved in the inhibition of focal adhesion formation, cell spread, and migration, as well as the inhibition of growth factor-stimulated MAPK signaling, a failed or altered PTEN expression can result in aberrant cell growth and apoptotic escape (reviewed in [76]).

Microsatellite instability (MSI) [85], specific mutations of K-ras [86], and *β*-catenin genes [87] are other genetic alterations in endometrioid endometrial cancer. Microsatellites are short segments of repetitive DNA bases scattered throughout the genome predominantly found in noncoding DNA. MSI, reported in 20% of sporadic endometrioid endometrial cancers, is caused by inactivation of any number of intranuclear proteins comprising the mismatch repair system, leading to an accumulation of structural changes in coding and noncoding repetitive elements of many genes [85]. Higher rates of mutations in the PTEN gene have been described in tumors displaying MSI as opposed to those that do not, suggesting that PTEN could be a target for mutations in a deficient DNA repair setting [88].

*β*-catenin, a component of the E-cadherin unit of proteins, plays an important role to cell differentiation, maintenance of normal tissue architecture, and to signal transduction. It also acts as a downstream transcriptional activator in the Wnt signal transduction pathway. These mutations result in stabilization of protein that resists degradation through the ubiquitin-proteasome pathway, leading to cytoplasmic and nuclear accumulation and constitutive target gene activity (reviewed in [76, 89, 90]).

Mutations in p53 are present in about 90% of tumors and constitute the most common genetic alterations in type 2 serous carcinomas [86]. After DNA damage, nuclear p53 accumulates and causes cell cycle arrest by inhibiting cyclin-D1 phosphorylation of the Rb gene, thereby promoting apoptosis [89]. Mutated p53 results in a nonfunctional protein that accumulates in the cell and acts as a double negative inhibitor of the wild-type p53, leading to propagation of aberrant cells. It has been suggested that mutation in one allele occurs during early development of serous carcinoma, and loss of the second normal allele occurs late in the progression to carcinoma. Other frequent genetic alterations in type 2 endometrial cancers include inactivation of p16 and overexpression of HER-2/neu [89]. Inactivation of p16 tumor suppressor gene, that encodes for a cell cycle regulatory protein, leads to uncontrolled cell growth and has been identified in 45% of serous carcinomas and some clear cell cancers (reviewed in [76]). HER-2/neu is an oncogene that codes for a transmembrane receptor tyrosine kinase involved in cell signaling. HER-2 overexpression and gene amplification have been found in 45% and 70% of serous carcinomas, respectively [91].

### 4.2. Molecularly Targeted Therapy

#### 4.2.1. mTOR Inhibitors

The activation of the PI3K/AKT/mTOR signalling pathway triggered by the loss of function of PTEN gene suggests a therapeutic role for the mammalian target of rapamycin (mTOR) inhibition. Chemotherapy-naïve endometrial cancer patients treated with temsirolimus, an mTOR inhibitor, achieved a preliminary response rate of 26% according to the National Cancer Institute of Canada; this result was not correlated to PTEN status as evaluated by immunohistochemistry [92]. Preliminary studies of other mTOR inhibitors, everolimus and AP2357, have shown clinical responses mainly in the form of stable disease (8 of 15 and 7 of 19 women, resp.) [93–95]. A phase II trial of temsirolimus in heavily pretreated patients with endometrial cancer, recently completed by the NCIC, reported a 7% partial response rate and a 44% stable disease rate [96]. It should be noted that the trials of everolimus and AP2357 were both in pretreated patients. Combinations of mTOR inhibitors with hormonal therapy, chemotherapy, or other targeted therapies such as epidermal growth factor receptor (EGFR) inhibitors and antiangiogenic agents have shown such promise, in the preclinical setting, that numerous trials are currently underway to develop and test such combinations; temsirolimus is being tested with topotecan, bevacizumab and progestin therapy (reviewed in [80]). It has been shown that exposure of endometrial cancer cell lines to an mTOR inhibitor increases progesterone mRNA expression and inhibits ER mRNA expression (reviewed in [80], [97]).

#### 4.2.2. Human Epidermal Growth Factor Receptor (EGFR) or HER Family Inhibition

**EGFR Inhibitors**

EGFR is commonly expressed in normal endometrium, but its overexpression in endometrial cancer is associated with advanced stage and poor prognosis [98–103]. Antagonists to EGFR include small molecule tyrosine kinase inhibitors (gefitinib, erlotinib, and lapatinib) and the anti-EGFR monoclonal antibody cetuximab. The use of erlotinib in women with recurrent and metastatic endometrial cancer was not promising with only 1 partial response among 27 women [104]. A phase II clinical trial of cetuximab in recurrent endometrial cancer is still ongoing. It is hoped that other new therapies will succeed in targeting specific known molecular defects in endometrial cancer, making significant headway in the prognosis of women with metastatic disease. Meanwhile, there is a need for an expedient second-line treatment, and clinical trials should be encouraged.

**Trastuzumab**

HER-2 amplification or overexpression has been demonstrated and linked to prognosis in endometrial cancer as well as in many other cancer types [105, 106]. HER-2/neu overexpression and gene amplification were found in about 20%–30% of serous carcinomas [91]. It is worth mentioning that in most series overexpression is more common than amplification. Trastuzumab is a monoclonal antibody to the extracellular domain of the HER-2 protein. Although HER-2 overexpression observed in serous carcinoma of the uterus provides a strong biologic rationale for the use of trastuzumab in the treatment of this malignancy, a GOG study examining the use of trastuzumab in women with HER-2 positive endometrial cancer did not report any activity [107].

#### 4.2.3. Angiogenesis Inhibition

It has been recognised that VEGF is key to tumour angiogenesis and progression representing the cornerstone of successful antineoplastic treatments. Increased levels of VEGF in endometrial cancer have been correlated with poor outcome. Preclinical models demonstrate the effectiveness of bevacizumab in combination with chemotherapy against endometrial cancer cell lines [108, 109]. Bevacizumab, a recombinant humanized immunoglobulin monoclonal antibody to vascular endothelial growth factor (VEGF), has proved to be effective and well tolerated in a number of malignancies. A small, retrospective study reviewed 10 patients with recurrent uterine neoplasms treated with bevacizumab. Two patients responded to treatment and the disease was stabilized in three patients [110]. A GOG phase II trial of single-agent bevacizumab in metastatic endometrial cancer has recently been completed, the results of which should soon be announced (GOG 229-E). VEGF-Trap is a recombinantly produced fusion protein consisting of human VEGF receptor extracellular domains fused to the Fc portion of a human immunoglobulin *γ* (IgG). It functions as a decoy receptor preventing the VEGF ligand from interacting with its ligand. A GOG phase II trial of VEGF trap in metastatic endometrial cancer is still in progress (GOG 229-F) (reviewed in [80]). A phase II trial of sorafenib, a tyrosine kinase inhibitor with antiangiogenic activity, has been completed in the National Cancer Institute's phase II network (reviewed in [80]). Preliminary results were not encouraging. A phase II trial of a second antiangiogenic tyrosine kinase inhibitor, sunitinib, is underway [111].

#### 4.2.4. Fibroblast Growth Factor Receptor 2 Inhibition

Fibroblast growth factor receptor 2 (FGFR2) is regulated on the basis of the balance of FGFs, heparan-sulfate proteoglycans, FGFR2 isoforms, endogenous inhibitors, and microRNAs [112]. The recent identification of activating mutations in FGFR2 in endometrial tumors has generated a new avenue for the development of targeted therapeutic agents [113, 114]. The majority of the mutations identified are identical to germline mutations in FGFR2 and FGFR3 that cause craniosynostosis and hypochondroplasia syndromes and result in both ligand-independent and ligand-dependent receptor activation [115]. Mutations that predominantly occur in the endometrioid subtype of endometrial cancer (16%) are mutually exclusive with KRAS mutation but occur in the presence of PTEN abrogation [116, 117]. In vitro studies have shown that endometrial cancer cell lines with activating FGFR2 mutations are selectively sensitive to a pan-FGFR inhibitor, PD173074 [113]. Oral administration of AZD2171 or Ki23057 inhibits in vivo proliferation of cancer cells with aberrant FGFR2 activation in rodent therapeutic models [112]. Several agents with activity against FGFRs are currently in clinical trials. Among PD173074, SU5402, and AZD2171 functioning as FGFR inhibitors, AZD2171 is the most promising anticancer drug [114]. Investigation of these agents in endometrial cancer patients with activating FGFR2 mutations is warranted [113].

## 5. Claudines

Epithelial receptors for clostridium perfringens enterotoxin (CPE), also known as claudines, may well prove to be the next target therapy for endometrial cancer, especially against aggressive disease variants. It has been shown that papillary-serous neoplasms overexpress claudines-1,-3 and -4 [118–120], while clear-cell ones overexpress claudines -3, and -4 [119]. Overexpression of claudines-3 and -4 could in part explain the aggressive behaviour of these histologies [119, 121] suggesting their potential as useful biomarkers or targets for type specific treatment.

## 6. Conclusion

Endometrial cancer is the most common gynaecological cancer in western countries. Although radiotherapy remains the cornerstone of postoperative management, accumulating data show that adjuvant chemotherapy may display promising results after staging surgery. Unfortunately, the prognosis of patients with metastatic disease remains disappointing with only-one year survival commonly reported despite treatment efforts. Progestins remain an effective option, especially for those patients with low-grade estrogen and/or progesterone receptor positive disease. Chemotherapy comprising paclitaxel, doxorubicin, and cisplatin is beneficial for patients with advanced or metastatic disease after radical surgery and potentially for patients with early-stage disease and high-risk factors. Toxicity is a concern, in which the development of less toxic regimens such as the combination of paclitaxel with carboplatin may diminish. In women with multiple medical comorbidities, single-agent chemotherapy may be better tolerated and still yield acceptable results. A better understanding of the molecular aspects of endometrial cancer biology has allowed clinical research to develop effective and targeted antineoplastic agents (everolimus, temsirolimus, gefitinib, erlotinib, cetuximab, trastuzumab, bevacizumab, sorafenib). Although targeted therapy is in general less toxic than chemotherapy, its use may be accompanied in some instances by considerable toxicity. Continued investigation into the molecular pathways of endometrial cancer development and progression will enhance existing knowledge of this disease process promoting the discovery of novel, superior treatment options for patients.

## Figures and Tables

**Table 1:** Trials on adjuvant treatment for endometrial cancer.

Author(s)	Setting	Pts	Treatment arms	5-year PFS^1^	5-year OS^2^	Comments
	(Stage)	(No.)		(%)	(%)	
Randall et al. [49]	III-IV	396	WAI^3^	38	42	Treatment related deaths AP: 8 (4%), WAI: 5 (2%)
	(optimally debulked)		AP^4^	50	55	
Maggi et al. [50]	ICG3-III	345	External beam	63	69	
			XRT^5^			
			CAP^6^	63	66	
Susumu et al. [52]	>50% myometrial invasion	385	Pelvic XRT	83.5	85.3	Superiority of CAP in high/intermediate risk (stICG3-IIIA) patients
			CAP	81.8	86.7	
Hogberg et al. [53]	IC-IIIC	367	Relvic XRT +/− BT^7^	75	NR^8^	
	(confined to pelvis)		Pelvic RT +/− BT + Cx^9^	82	NR	
Kuoppala et al. [51]	IAG3-IIIA	156	Pelvic XRT	18+ months	84.7	Intestinal complications demanding surgery
			Pelvic XRT + CEP^10^	25+ months	82.1	XRT: 2 (2.7%), Pelvic XRT + CEP: 8 (9.5%)

^1^PFS, progression-free survival; ^2^OS, overall survival; ^3^WAI, whole abdominal irradiation; ^4^AP, doxorubicin and cisplatin; ^5^XRT, irradiation; ^6^CAP, cyclophosphamide, doxorubicin and cisplatin; ^7^BT, vaginal brachytherapy; ^8^NR, not reported; ^9^Cx, chemotherapy with AP or paclitaxel, epirubicin and carboplatin or paclitaxel and carboplatin; ^10^CEP, cyclophosphamide, epirubicin and cisplatin.

**Table 2:** Types of endometrial cancer according to the Bokhman model and correlations with clinicopathological and molecular characteristics.

Characteristics	Type I tumors	Type II tumors
*Clinicopathological*		
Incidence	~80%	~20%
Age at initial diagnosis	Pre/peri-menopausal	Postmenopausal
Histology	Endometrioid	Non-endometrioid (predominantly serous and clear cell)
Grade	Usually low	Usually high
Premalignant phase	Atypical hyperplasia	Glandular dysplasia (for serous tumours)
Predisposing factors	Obesity, prolonged estrogen exposure	
ER, PgR	>90%	0–31%
*Molecular*		
HER-2/neu (overexpression)	3%	18%
EGFR expression	46%	34%
P53 mutations	5–10%	80–90%
Ploidy	67% diploid	45% diploid
PTEN (loss of function through deletion or mutation)	50–80%	10–11%
P16 inactivation	10%	40%
K-ras (mutational activation)	13–26%	0–10%
E-cadherin (reduced or non expression)	10–20%	62–87%
*β*-catenin CTNNB1 (gain of function mutation)	25–38%	Rare
